# Extracellular Vesicles: Emerging Modulators of Cancer Drug Resistance

**DOI:** 10.3390/cancers13040749

**Published:** 2021-02-11

**Authors:** Fabrizio Fontana, Emanuela Carollo, Genevieve E. Melling, David R. F. Carter

**Affiliations:** 1 Department of Pharmacological and Biomolecular Sciences, Università degli Studi di Milano, via Balzaretti 9, 20133 Milan, Italy; 2 Department of Biological and Medical Sciences, Oxford Brookes University, Gipsy Lane, Oxford OX3 0BP, UK; manu.carollo-2017@brookes.ac.uk (E.C.); g.e.melling@bham.ac.uk (G.E.M.); 3 Institute of Clinical Sciences, College of Medical and Dental Sciences, University of Birmingham, Birmingham B15 2TT, UK

**Keywords:** extracellular vesicles, cancer drug resistance, chemotherapy, MDR transporters, miRNAs, tumor microenvironment, cancer stem cells

## Abstract

**Simple Summary:**

Drug resistance still represents the main reason for therapy failure in cancer patients. In the last decade, extracellular vesicles (EVs), a heterogeneous group of particles implicated in cell-to-cell communication, have been shown to substantially contribute to this phenomenon. This review summarizes the molecular mechanisms underlying the EV-mediated development of chemoresistance, shedding light on the potential role of these vesicles as both diagnostic/prognostic markers and therapeutic targets.

**Abstract:**

Extracellular vesicles (EVs) have recently emerged as crucial modulators of cancer drug resistance. Indeed, it has been shown that they can directly sequester anti-tumor drugs, decreasing their effective concentration at target sites. Moreover, they facilitate the horizontal transfer of specific bioactive cargoes able to regulate proliferative, apoptotic, and stemness programs in recipient cells, potentially conferring a resistant phenotype to drug-sensitive cancer cells. Finally, EVs can mediate the communication between the tumor and both stromal and immune cells within the microenvironment, promoting treatment escape. In this context, clarifying the EV-driven resistance mechanisms might improve not only tumor diagnosis and prognosis but also therapeutic outcomes. Detailed cellular and molecular events occurring during the development of EV-mediated cancer drug resistance are described in this review article.

## 1. Introduction

Extracellular vesicles (EVs) are a heterogeneous population of nano-sized, membrane-delineated vesicles involved in cell-to-cell communication [1]. They can vary in size, function, and biogenesis, and are mostly classified as: exosomes, 30–100 nm particles which originate from the endosomal compartment and are secreted upon fusion of multivesicular bodies (MVBs) with the cell membrane; microvesicles, which are 100 nm–1 μm in diameter and formed via outward budding and fission of the cell membrane; apoptotic bodies, ranging from 50 nm to 5 µm and released as blebs of cells undergoing apoptosis [2]. EVs can be found in various body fluids, including blood, urine, and saliva, and can be used for the transfer of DNA, mRNA, microRNAs (miRNAs), long non-coding RNAs (lncRNAs), and proteins from the originating cells to both neighboring and distant cells [3]. They have been shown to modulate different tumorigenic processes, such as cancer proliferation, migration, and angiogenesis, and are implicated not only in the horizontal transmission of biological cargo but also in the interactions between malignant and non-malignant cells in the tumor microenvironment [4,5,6]. Based on these observations, accumulating evidence suggests that they can play a crucial role in the development of cancer drug resistance. This review is aimed at summarizing the recent findings about the involvement of EVs in the onset of a drug-resistant phenotype in cancer.

## 2. EVs and Cancer Drug Resistance

**Figure 1:**
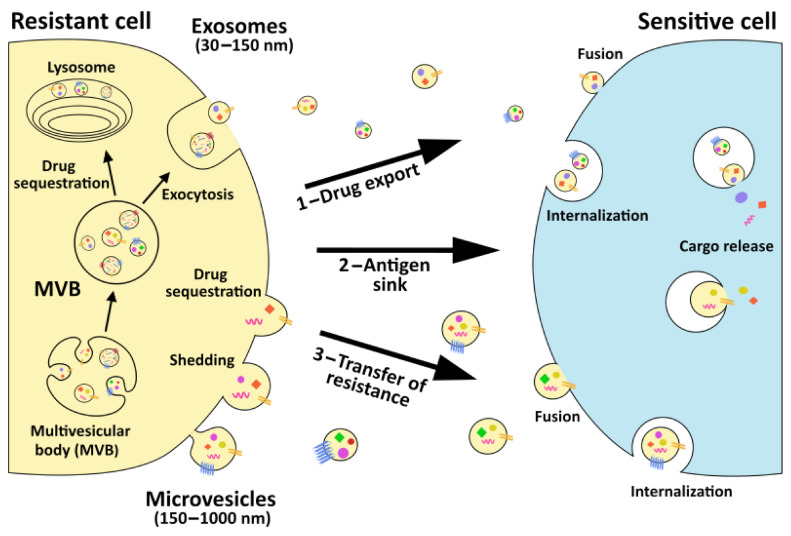
Graphical representation of extracellular vesicle (EV) roles in cancer drug resistance. Both microvesicles and exosomes can facilitate drug sequestration by directly loading and exporting anti-tumor agents from cancer cells (step 1) or by binding on their surface therapeutic antibodies in the extracellular space (step 2). Once released, EVs can also be taken up by recipient cells and release their cargo. Cargo release can affect recipient cell phenotype, modifying their sensitivity to specific drugs (step 3).

The main barrier to the development of an effective anti-cancer strategy is represented by the acquisition of drug resistance by tumor cells, a phenomenon that is responsible for up to 90% of cancer-related deaths [7]. Indeed, despite being initially susceptible to standard therapies, tumor cells frequently become tolerant to current treatments via different mechanisms able to impair drug efficacy, including reduced drug absorption, altered drug metabolism, drug target mutation or expression modification, cell death suppression, increased DNA repair, gene amplification, and epigenetic changes [7,8]. Among them, the EV-mediated sequestration of anti-tumor agents and transfer of drug efflux pumps, as well as the EV-related transmission of pro-survival, anti-apoptotic, and stemness-associated genetic and protein cargo appear to crucially contribute to the emergence of chemoresistance (Figure 1).

### 2.1. EVs and Drug Sequestration at Intracellular and Extracellular Levels

Regardless of the administration route, anti-cancer drugs are designed to effectively reach the tumor site, where cell permeability or transport across the plasma membrane represent key factors in determining drug uptake and treatment success. Interestingly, EVs can be utilized by cancer cells to promote chemoresistance through direct drug load and expulsion. In this regard, a correlation between vesicle shedding-associated gene expression and drug resistance was first reported by Shedden et al. in a panel of 60 different tumor cell lines [9]. In particular, in MCF7 breast cancer cells and in SU-DHL-4 and Balm3arge B-cell lymphoma cells, doxorubicin was found to be physically incorporated into EVs and excreted into the media [9]. Similarly, melanoma cells could survive cisplatin treatment via an extracellular acidification-induced increase in EV secretion and subsequent export of the chemotherapeutic agent into these vesicles [10]. This was also confirmed in an in vitro model of cisplatin-unresponsive ovarian carcinoma, where EVs from resistant cells not only contained multidrug resistance-associated protein 2 (MRP-2) but also the copper-transporting P-type ATPases ATP7A and ATP7B [11]. In this respect, it should be noted that ATP-binding cassettes (ABC) can localize to the limiting membranes of EV-like structures and facilitate drug sequestration. This applies to ABCG2, whose expression in breast cancer is highly confined to cell–cell attachment zones able to generate mitoxantrone-loaded vesicles [12]. On the other hand, in daunorubicin-tolerant leukemia cell lines, high levels of ABCA3 have been observed on the surface of MVBs, in which the chemotherapeutic agent is efficiently internalized [13].

Intriguingly, EVs can also mediate drug removal from the extracellular space. For example, they can decrease the extracellular levels of anti-cancer therapeutic antibodies by displaying specific antigens on their surface. Indeed, B-cell lymphoma EVs have been reported to carry the cluster of differentiation (CD)-20 receptor, thus binding to the anti-CD20 chimeric antibody rituximab and protecting target cells from its attack [14]. In an in vitro model of breast cancer, the human epidermal growth factor receptor-2 (HER2) has been demonstrated to be overexpressed on the EV membrane, acting as a bait for the monoclonal antibody Herceptin [15]. EV-mediated reduction in drug availability has also been observed in epithelial cell adhesion molecule (EpCam)-positive breast cancer cells treated with the EpCam-specific antibody C215, suggesting a correlation between EV release and tumor progression [16].

Similar to the above reported antibody-inactivating tumor antigens, decoy receptors can be exposed on the EV surface for anti-cancer drug binding. This is the case of tumor necrosis factor-related apoptosis inducing ligand (TRAIL): in a recent study by Setroikromo et al., colorectal tumor cells have been shown to secrete DR5-coated EVs able to sequester the pro-apoptotic ligand, thus reducing drug sensitivity [17].

### 2.2. EVs and Acquisition of a Multidrug Resistant Phenotype

In addition to direct drug sequestration, EVs can be exploited by tumor cells to transmit chemoresistance via horizontal transfer of drug efflux pumps. These proteins utilize ATP for active removal of drugs from the cytoplasm, preventing their accumulation in cancer cells [18,19].

The multidrug resistance protein 1 gene (*MDR1* or *ABCB1*) encodes for the main drug transporter P-gp, whose expression has been observed in the majority of tumors with MDR phenotype and correlated to tolerance to at least 20 different chemotherapeutic agents [20]. A significant amount of research has highlighted that P-gp can be transferred from drug-resistant to drug-sensitive cancer cells by circulating EVs, promoting acquired therapy resistance both in vitro and in vivo [21,22,23]. Mechanistically, functional P-gp is encapsulated into the vesicular membrane and carried to recipient cells, which expose it on their surface [22]. This has been observed, for instance, in several in vitro models of breast cancer, where docetaxel- and doxorubicin-sensitive cells were able to acquire an MDR phenotype upon exposure to P-gp-carrying EVs isolated from their resistant counterparts. In particular, the enhanced levels of P-gp found in recipient cells were proportional to the number of EVs shed from donor cells [24]. Similarly, taxane resistance was conferred to prostate and ovarian cancer cells by their resistant variants via exosomal P-gp transport, while osteosarcoma cells were reported to spread their ability to escape doxorubicin treatment by EV-related transmission of MDR-1 mRNA [25]. In vivo experiments confirmed this exosomal P-gp transfer in adriamycin-refractory breast cancer xenografts as well as in colchicine-unresponsive neuroblastoma-bearing mice, even evidencing a more efficient MDR transmission in physiological conditions than in cell cultures [21].

MDR development has also been attributed to multidrug resistance-associated protein 1 (MRP1 or ABCC1) activation [26]. Recently, Lu et al. have demonstrated the EV-mediated dissemination of functional MRP1 in leukemia cells. Notably, they have also shown a significant ability for EVs released from cells with a P-gp dominant resistance profile to re-template a pre-existing MRP1 dominant profile in recipient cells [27].

Other drug efflux exporters, such as ABCG2 or ABCA3, have been reported to be horizontally transferred through EVs, favoring the excretion of a variety of cytotoxic agents and thus, modulating chemoresistance in tumor cells [14,28].

It should be outlined that the transfer of MDR transporters alone cannot explain the long-lasting effects described in the literature [21,23]. In this regard, it has been recently suggested that the prolonged induction of ABC protein expression observed in EV-recipient cells may be caused by the uptake of MDR-associated mRNAs and miRNAs. Indeed, transcription of specific EV-delivered mRNAs appears to be implicated in the activation of nuclear factor kappa B (NF-κB), which is known to upregulate MDR1 expression [29]. In parallel, both miR-451 and miR-27a contained in drug resistant cell-derived EVs are able to induce P-gp expression, explaining the emergence of long-term chemoresistance [23,30].

### 2.3. EVs and Horizontal Transfer of Pro-Survival Proteins and RNAs

EVs can elicit pro-survival and anti-apoptotic signals in tumor cells, leading to resistance to a wide spectrum of chemotherapeutics.

The PI3K/AKT pathway is one of the main oncogenic cascades implicated in cancer cell proliferation [31,32]. Activation of this axis and consequent development of in vitro and in vivo tolerance to sorafenib has been found in hepatocellular carcinoma cells following EV-regulated delivery of hepatocyte growth factor (HGF) [33]. Likewise, PI3K/AKT induction resulting from EV-mediated transfer of platelet-derived growth factor receptor-beta (PDGFR-β) has been shown to be responsible for melanoma resistance to the BRAF inhibitor PLX4720 [34]. Finally, triple negative breast cancer cell lines resistant to both doxorubicin and docetaxel have been demonstrated to release EVs able to boost PI3K/AKT signaling in non-malignant breast cells, suggesting that they may contain upstream regulators of this pathway [35]. Collectively, these results confirm the centrality of the PI3K/AKT cascade in tumor aggressiveness, supporting the use of novel AKT inhibitors, such as afuresertib, ipatasertib, and perifosine, for combination therapy.

Increased levels of survivin, a protein belonging to the family of the inhibitors of apoptosis (IAP), have been reported in EVs isolated from various tumor types, including cervical and prostate cancer, where it enhances protection against genotoxic stresses and proton irradiation [36,37,38]. More recently, treatment of MDA-MB-231 breast cancer cells with paclitaxel has been shown to trigger the secretion of survivin-enriched EVs that can modulate the resistance of the tumor not only to the drug itself but also to other stressful conditions, such as serum starvation [39]. Despite still being unclear, the mechanism responsible for the vesicular enrichment of survivin appears to be rather specific. Indeed, this protein is preferentially expressed in exosomes, while being absent in microvesicles [37,38,39]: this raises the intriguing possibility that the two EV subtypes might mediate distinct biological effects in cancer.

Radiation-induced DNA damage can be prevented by EV uptake in cancer cells. Indeed, irradiated breast cancer cells can release EVs able to activate checkpoint kinase 1 (Chk1), histone H2AX, and ataxia telangiectasia mutated (ATM) in recipient cells, thus triggering DNA repair responses [40]. Similarly, EVs from head and neck cancer can increase radioresistance in neighboring cells by triggering DNA double strand break-correcting processes [41,42]. EVs can further enhance survival to radiation by conferring an invasive phenotype to cancer cells and thus, facilitating their migration from the irradiated area, as observed in an in vitro model of glioblastoma [43]. Of note, these studies also indicate that radiation increases exosomal release and uptake, confirming the crucial role of EVs as a communication tool in the acute radiation stress response [40,41,42,43]. In this setting, EV-targeted strategies may provide a novel approach to prevent tumor progression and improve radiotherapy outcome.

Tumor cells have often limited access to oxygen and nutrients, thereby being subjected to hypoxia [44]. In this context, ovarian cancer cells cultured in hypoxic conditions have been found to significantly increase EV release via upregulation of Rab27a and inactivation of Rab7, LAMP1/2, and NEU-1 [45]. More importantly, hypoxia-induced EVs contributed to the cisplatin resistance of these cells by spreading the oncogenic transcription factor STAT3, with inhibition of this protein resulting in decreased EV secretion and reduced cell proliferation and colony formation after chemotherapy [45]. A recent in vivo study has further highlighted the role of STAT3 in the vesicular transfer of 5-fluorouracil tolerance in colorectal cancer [46]. More investigations are needed to validate the clinical use of STAT3-containing EVs as a therapeutic target and biomarker in both ovarian and colon carcinomas.

As with most malignancies, non-small-cell lung cancer (NSCLC) consists of both epithelial and mesenchymal tumor cells, with the latter thought to promote resistance to standard treatments [47]. By using a human bronchial epithelial cell model in which parental cells are forced to acquire a mesenchymal phenotype through oncogenic manipulation, Lobb et al. have demonstrated that EVs secreted by mesenchymal, oncogenically transformed lung cells can transfer chemoresistance to epithelial cells via the transmission of ZEB1 mRNA [48]. Hence, this research reveals a novel mechanism by which phenotypic changes can occur in the primary, heterogeneous tumor mass through vesicular communication.

Chloride intracellular channel 1 (CLIC1) is a 241-amino acid ion channel known to contribute to the malignant transformation of gastric cells [49,50]. Fascinatingly, the vesicular transfer of this protein has been found to induce vincristine resistance in gastric cancer, an effect associated with the upregulation of P-gp and Bcl-2 [51].

Glutathione S-transferases (GSTs) are a class of enzymes crucially implicated in xenobiotic detoxification by catalyzing the conjugation of different electrophilic/hydrophobic molecules with reduced glutathione [52]. The EV-mediated transmission of GSTP1 mRNA from chemoresistant to chemosensitive breast cancer cells has been demonstrated to increase the survival of the latter to adriamycin treatment [53]. More recently, a proteomic study has also evidenced the enrichment of GSTP1 in EVs from 5-fluorouracil-tolerant colon cancer cells [46]. Although many factors are implicated in drug resistance, these findings on GSTP1 open interesting possibilities for clinical application in cancer prognosis.

In addition to genetic factors, accumulating evidence suggests that epigenetic changes contribute to chemoresistance [54]. Aberrant genomic DNA methylation pattern and histone modifications finely regulate gene expression, thus affecting cancer progression and recurrence. Three main DNA methyltransferases (DNMTs) exist in mammals: DNMT1, 3A, and 3B. DNMT1 is the one controlling genome-wide methylation during DNA replication and repair, while 3A and 3B catalyze de novo methylation patterns [55]. Using an ovarian cancer xenograft mouse model, Cao et al. have shown that vesicular DNMT1 mRNA is involved in cisplatin resistance [56].

MiRNAs are endogenous, non-coding RNAs that are around 22 nucleotides in length and can stimulate both transcriptional and translational arrest, thereby acting as either oncosuppressors or oncogenes, depending on the specific cancer type [57]. EVs carrying various anti-apoptotic miRNAs have been implicated in the transfer of chemoresistance to sensitive cells in several human tumor models, such as lung, breast, colon, and pancreatic cancer, leukemia, melanoma, and glioblastoma. Moreover, EVs have been shown to increase the therapy resistance of recipient cells by decreasing intracellular levels of tumor suppressive miRNAs. An updated list of the miRNAs known to be deregulated in cancer EVs is presented in Table 1; among them, altered levels of circulating miR31-5p, miR-155, miR-425-3p, miR-744, and miR-1238 have been found in unresponsive patients undergoing different anti-cancer therapies [58,59,60,61,62].

LncRNAs are transcripts with lengths exceeding 200 nucleotides that are not translated into proteins [63]. Similar to miRNAs, they have been shown to play a key role in EV-mediated chemoresistance in several tumor cell lines (Table 2). Remarkably, the clinical relevance of the vesicular lncRNAs ARSR and HOTTIP has been recently confirmed by their detection in the serum of renal cell carcinoma and gastric cancer patients exhibiting tolerance to sunitinib and cisplatin, respectively [64,65].

**Table 1:** Vesicular miRNAs involved in cancer drug resistance.

miRNA	Tumor	Drug	Ref
miR-19b	Colorectal cancer, leukemia	Oxaliplatin, daunorubicin	[66,67]
miR-20a	Leukemia	Daunorubicin	[67]
miR-21	Oral squamous cell carcinoma, leukemia, breast cancer	Cisplatin, multidrug	[68,69]
miR-34a	Colon cancer, prostate cancer	5-FU, docetaxel	[70,71]
miR-31-5p	Renal cell carcinoma	Sorafenib	[58]
miR-96	Lung cancer	Cisplatin	[72]
miR-100-5p	Lung cancer	Cisplatin	[73]
miR-134	Breast cancer	Multidrug	[74]
miR-145	Colon cancer	5-FU	[70]
miR-155	Breast cancer, lung cancer	Doxorubicin, paclitaxel, gemcitabine	[59,75,76]
miR-155-5p	Breast cancer	Docetaxel, doxorubicin	[35]
miR-211-5p	Melanoma	Vemurafenib	[77]
miR-221/222	Breast cancer	Tamoxifen	[78]
miR-222	Breast cancer	Adriamycin, docetaxel	[79,80]
miR-222-3p	Lung cancer	Gemcitabine	[81]
miR-365	Leukemia	Imatinib	[82]
miR-425-3p	Lung cancer	Cisplatin	[60,83]
miR-744	Hepatocellular carcinoma	Sorafenib	[61]
miR-761	Synovial sarcoma	Pazopanib	[84]
miR-1238	Glioblastoma	Temozolomide	[62]
miR-1246	Breast cancer	Multidrug	[85]

**Table 2:** Vesicular lncRNAs implicated in cancer drug resistance.

lncRNA	Tumor	Drug	Ref
linc-AGAP2-AS1	Breast cancer	Trastuzumab	[86]
lncARSR	Renal cell carcinoma	Sunitinib	[64]
lncHNF1A-AS1	Cervical cancer	Cisplatin	[87]
lncHOTTIP	Gastric cancer	Cisplatin	[65]
linc-ROR	Hepatocellular carcinoma	Sorafenib	[88]
linc-SBF2-AS1	Glioblastoma	Temozolomide	[89]
lincSNHG14	Breast cancer	Trastuzumab	[90]
linc-VLDLR	Hepatocellular carcinoma, esophageal cancer	Multidrug	[91,92]

### 2.4. EVs and Interactions with Stromal and Immune Cells in the Tumor Microenvironment

Cancer drug resistance is determined not only by cancer cells themselves but also by the non-malignant cells within the tumor microenvironment, and recent findings have highlighted the crucial role of EVs in the regulation of this crosstalk.

Cancer-associated fibroblasts (CAFs) and adipocytes (CAAs), as well as bone marrow mesenchymal stem cells (MSCs), make up the tumor stroma and have been increasingly acknowledged as major contributors to the development of chemoresistance [93,94]. Indeed, by secreting EVs containing annexin A6, CAFs have been found to stabilize β1 integrin and upregulate focal adhesion kinase (FAK)-Yes-associated protein (YAP) expression in gastric cancer cells, increasing their survival after treatment with cisplatin [95]. In addition, several EV-encapsulated miRNAs and lncRNAs, such as miR-146, miR-27a, miR-106b, miR-196a, and lncCCAL, are spread from fibroblasts to associated tumors, thus affecting cancer sensitivity to a wide range of chemotherapeutics [96,97,98,99,100]. Likewise, the exosomal transfer of miR-21 from CAAs to ovarian cancer cells has been reported to reduce paclitaxel-induced apoptosis via downregulation of apoptotic peptidase activating factor (APAF1) mRNA [101]. Moreover, human MSC-derived EVs have been shown to promote resistance of gastric cancer cells to 5-fluorouracil by activating the CaM-Ks/Raf/MEK/ERK cascade both in vivo and ex vivo [102]. Similar results have been obtained in MSC EV-treated multiple myeloma cells, where upregulation of proteasome 20S subunit alpha 3 (PSMA), lncPSMA3-AS1, and Bcl-2, as well as reduced caspase-3 and -9 cleavage and c-Jun N-terminal Kinase (JNK) phosphorylation were observed, resulting in bortezomib tolerance [103,104].

It is now clear that macrophages are recruited by tumor cells to mediate mechanisms of drug resistance [105]. Binenbaum et al. have recently demonstrated that the transmission of miR-365 in macrophage-derived EVs confers gemcitabine resistance to pancreatic adenocarcinoma cells in vitro and in vivo [106]. Similarly, exosomal miR-21 can be delivered from macrophages to gastric cancer cells, where it prevents cisplatin-triggered apoptosis via inhibition of PTEN and subsequent activation of the PI3K/AKT pathway [107]. Finally, EVs shed from hypoxic macrophages transfer miR-223 to ovarian carcinoma cells to elicit a chemoresistant phenotype [108]. Interestingly, the EV-mediated crosstalk between cancer and innate immune cells is bidirectional: different chemotherapeutics, such as melphalan, carfilzomib, and bortezomib, dramatically stimulate the release of heparanase-rich EVs in myeloma cells, and the exposure of macrophages to these vesicles increases the secretion of pro-tumor TNF-α [109]. More importantly, Callaghundla et al. have shown that the “educational” process elicited by neuroblastoma cells on human monocytes through the secretion of vesicular miR-21 not only leads to a M2 polarization of the immune cells but also to a polarized monocyte-mediated TLR8 and NF-кB-dependent upregulation of miR-155 in neuroblastoma cells themselves [110].

More recently, EVs have been found to contribute to the development of immunotherapy resistance. Indeed, despite being largely used, treatment with monoclonal antibodies that block immune regulatory checkpoint receptors or ligands, such as programmed cell death protein 1 (PD-1) or programmed death-ligand 1 (PD-L1) inhibitors, is not always followed by effective responses in cancer patients [111,112]. This may be due to the presence of PD-L1 on tumor-derived EVs, which can capture the corresponding immunotherapeutic antibody on their surface, allowing the tumor to engage PD-1 on T cells. Such mechanism has been described in an in vitro model of glioblastoma, where cancer-released EVs have been shown to express PD-L1 and inhibit T cell proliferation as well as antigen-specific T cell responses [113]. More importantly, the early exposure of PD-L1 on the EV surface has been proposed as a novel parameter to classify melanoma patients as anti-PD-1 therapy responders or resistant [114]. Regarding immune surveillance evasion, it should also be emphasized that tumor-secreted EVs can directly impair CD8+ T lymphocyte function by carrying the pro-apoptotic Fas Ligand (FasL) and the suppressive galectin-1 and -9, both in vitro and in vivo [115,116,117,118,119,120,121]. In addition, they can alter the adaptive immune responses by promoting regulatory T cell proliferation via TGF-β1 to the detriment of other T cell subsets [122,123,124] or by inhibiting the differentiation of bone marrow progenitor cells into dendritic cells [125], with consequent block of tumor antigen presentation and further T cell activation.

### 2.5. EVs and Modulation of Cancer Stem Cell-Like Features

Cancer stem cells (CSCs) are a small subpopulation of cancer cells inhabiting the tumor mass, known to orchestrate tumorigenesis and, more importantly, to mediate therapy resistance and tumor relapse [126,127]. Emerging evidence suggests that EVs are deeply involved in the modulation of the population equilibrium within the tumor, favoring the expansion of those cells deputed to therapy escape and cancer growth re-initiation. For instance, Kock et al. have demonstrated the EV-mediated horizontal transfer of Wingless-related integration site (Wnt) signaling-associated CSC traits in an in vitro model of diffuse large B cell lymphoma characterized by doxorubicin resistance [128]. In addition, resistance to proteasome inhibitors can be transmitted through EV-induced cell cycle arrest and enhanced stemness in leukemia cells [129]. Moreover, chemotherapy has been found to stimulate breast cancer cells to secrete multiple vesicular miRNAs, including miR-203a-3p, miR-195-5p, and miR-9-5p, which simultaneously inactivate the transcription factor one cut homeobox 2 (ONECUT2) and increase the expression of stemness-associated genes, such as *SOX2*, *OCT4*, *NANOG*, *SOX9*, and *NOTCH1*; inhibition of these miRNAs or restoration of ONECUT2 expression abolishes the CSC-stimulating effect of EVs from chemotherapy-treated cancer cells [130]. Overall, this evidence provides novel insights into how cancer plasticity may promote chemoresistance, highlighting that primary tumors are characterized by a self-organized infrastructure where the conversion of cell states is modulated by an EV-mediated intercellular communication.

With the aim of shedding some light on the role played by CSCs in the development of drug resistance, recent studies have led to the proposal of a new model for cancer cell dedifferentiation, in which the acquisition of stem cell-like features is finely orchestrated by the tumor microenvironment through vesicular transfer. Notably, breast cancer cells have also been shown to prime MSCs to release EVs containing distinct miRNAs, such as miR-222/223, which in turn promote tumor quiescence and dormancy, ultimately culminating in carboplatin resistance [131]. Furthermore, stroma-derived EVs have been reported to induce dedifferentiation of lung and breast cancer cells to a chemoresistant CSC-like phenotype via neurogenic locus notch homolog protein 3 (Notch3)/signal transducer and activator of transcription 1 (STAT1) signaling and interleukin-6 (IL-6), activin-A, and granulocyte colony stimulating factor (G-CSF), respectively [132,133]. Similar results have been obtained in OVCAR-5 ovarian carcinoma cells, where an enrichment in the drug-resistant EpCAM^+^CD45^+^ subpopulation has been observed after treatment with EVs secreted by the non-tumor cells of the ascitic fluid [134]; likewise, fibroblast-derived EVs stimulate growth and clonogenicity of colorectal CSCs (i.e., CD133+ and TOP-GFP+) upon treatment with oxaliplatin and 5-fluorouracil [135]. Finally, CAFs can promote colon cancer stemness and chemoresistance via vesicular transfer of lncH19, which activates the β-catenin pathway by acting as a competing endogenous RNA sponge for tumor-suppressive miR-141 [136]. Intriguingly, besides inducing a CSC-like phenotype in cancer cells, EVs from fibroblasts can reverse this dormant state by transferring mitochondrial DNA and promoting oxidative phosphorylation, thus facilitating disease recurrence and metastasis [137]. This has also been observed in an in vivo model of breast cancer, where differentially activated macrophages within the bone marrow stroma regulate the behavior of CSCs by either inducing or reversing dormancy via EV secretion [138]. Identification of these EV-regulated interactions opens a new branch of potential tumor therapies targeting the microenvironment. Further studies are expected to identify novel molecules to be administered in combination with conventional anti-cancer protocols to specifically abrogate these communication pathways.

### 2.6. EVs as Tools to Monitor Response to Cancer Treatment

The search for specific, reliable, and highly sensitive biomarkers to predict cancer progression and treatment response is an extremely challenging task that, if fulfilled, could revolutionize cancer care pathways, paving the way towards personalized medicine. Carcinoembryonic antigen (CEA), alpha-fetoprotein (AFP), carbohydrate antigen 125 (CA-125), and other serum markers are often characterized by low specificity and sensitivity and their expression can be affected by non-pathological conditions: both these factors make these indicators not ideal as a primary choice for cancer diagnosis and prognosis, highlighting the need for novel, valid tumor biosignatures [139].

The biological composition of EVs and their abundance in biofluids, especially in plasma, make them excellent candidates for monitoring patients’ response to cancer treatment. A potential breast cancer biomarker has been identified by Van Dommelen et al.: by conducting preliminary in vitro studies on A-431 cells, they found that cetuximab administration decreased the expression of EGFR in tumor-derived EVs, and the same changes were described in parental cells as well [140]. In addition, König et al. have shown that EV concentration is increased in the blood of breast cancer patients after neoadjuvant chemotherapy (NACT); in particular, treatment failure was observed in patients displaying high pre-NACT EV levels, while enhanced post-NACT EV concentrations were associated with a decrease in overall survival [141]. Vesicular PD-L1 protein and mRNA copy numbers could be used to monitor the response to pembrolizumab and nivolumab in melanoma and NSCLC: while immunotherapy-unresponsive patients have demonstrated high pre-treatment levels of PD-L1, PD-L1 mRNA declined in responsive patients [114,142]. Likewise, digital droplet PCR has been used to measure EV-related KRAS mutant allele frequency (MAF) in NACT-treated pancreatic cancer, evidencing a decrease in KRAS MAF in responsive patients with no disease progression [143]. Plasma-derived EV-associated KRAS mutations have also been found to drop after surgical resection of pancreatic ductal adenocarcinoma, suggesting that quantification of vesicular KRAS mutation could be adopted for the assessment of tumor burden and therapy response [144]. EVs can finally be used to monitor radiotherapy efficacy; Malla et al. have reported an increase in EV number in the serum of prostate cancer patients, with a further rise in post-radiation samples. Moreover, exosomal hsa-let-7a-5p and hsa-miR-21-5p were upregulated upon radiation, indicating their potential value as prognostic biomarkers [145].

### 2.7. Strategies to Overcome EV-Related Cancer Drug Resistance

As outlined in this review, drug resistance can be conferred through the EV-mediated sequestration of the drug itself or transfer of bioactive factors that promote drug expulsion or induce downstream desensitization. Therefore, a viable strategy to combat EV-related chemoresistance, which could be used in combination with therapy itself, is to inhibit EV communication.

Studies investigating EV-associated drug resistance have used a variety of techniques to suppress EV formation, release, uptake, or cargo transfer. Inhibiting EV biogenesis using GW4869 was able to prevent CAFs from releasing EVs capable of desensitizing pancreatic cancer cells towards gemcitabine [96]. GW4869 treatment could also block EV-mediated cisplatin desensitization due to the transfer of DNMTs [56]. Pharmacological inhibitors of EV uptake, including dynasore, amiloride, and heparin, have been used to increase cell sensitivity towards cisplatin treatment in vitro [146]. In addition, dynamin 2 and clathrin knockdown severely altered vincristine-induced EV-modulated transfer of ABCB1, thus preventing recipient oral epidermoid carcinoma cells from becoming chemoresistant [147]. However, not all EV communication is pro-tumorigenic, and given the high complexity and heterogeneity of vesicular biogenesis, there is still no valid strategy able to suppress the release and consequent uptake of the entire spectrum of particles. Moreover, the above agents often have off target effects due to the lack of specific anti-EV mechanisms (as summarized in [148]). Therefore, further work is needed to understand how to specifically target deleterious EV subtypes and delineate whether these strategies are effective in vivo.

Several reports have indicated that the functional transfer of EVs containing MDR transporters leads to the desensitization of cancer cells to chemotherapeutics; hence, reducing the proportion of these vesicular pumps might be a useful therapeutic option. Koch et al. have found that B-cell lymphoma sensitivity to doxorubicin can be increased by using an ABCA3 shRNA; interestingly, this genetic approach resulted not only in reduced EV-mediated drug load and expulsion but also in decreased EV spread [149]. Similarly, treatment of metastatic melanoma cells with a proton pump inhibitor lead to enhanced cisplatin response via a reduction in EV production [10]. Collectively, these results not only support the relevance of MDR transporters as direct genetic and pharmacological targets in cancer therapy but also point out that vesicular biogenesis is critically dependent on the expression of these proteins, thus opening the way to novel EV-targeting anti-tumor strategies.

A newly proposed method of altering EV communication for preventing chemoresistance is to entrap circulating decoy EVs responsible for the sequestration of active drugs. In this regard, a hemofiltration system has been recently developed to specifically remove circulating HER2-positive EVs in breast cancer patients, in order to increase the efficacy of Herceptin [150].

## 3. Conclusions and Future Perspectives

Most of the experimental evidence summarized herein demonstrates that EVs play a key role in favoring the emergence of cancer drug resistance through several mechanisms, including direct drug load and expulsion and transfer of pro-survival, anti-apoptotic, and stemness-associated genetic and protein cargo. In this context, EV profiling could be exploited in tumor prognosis, with EV-based liquid biopsies likely aiding in the prediction of response to therapies while avoiding invasive biopsy procedures. On the other hand, EV targeting has shown promise as an anti-cancer strategy aimed at overcoming chemoresistance. However, despite the scientific robustness, the majority of the studies described above have been conducted in vitro and to a lesser extent in vivo; therefore, further experiments should be performed in more physiologically relevant models to clarify the EV signaling network in perspective of a clinical application. Nevertheless, some scientific challenges, such as a deeper dissection of EV heterogeneity and the development of standardized isolation techniques, still need to be addressed to fully translate EV research into the clinical setting.

## Data Availability

No new data were created or analyzed in this study. Data sharing is not applicable to this article.
